# Negotiating ethnolinguistic identity in a multilingual society: social meaning and linguistic choice in Namibian German

**DOI:** 10.3389/fpsyg.2026.1819162

**Published:** 2026-05-29

**Authors:** Antje Sauermann, Britta Schulte, Heike Wiese

**Affiliations:** Department of German Studies and Linguistics, Humboldt University of Berlin, Berlin, Germany

**Keywords:** German, language attitudes, linguistic choice, Namibia, societal context

## Abstract

**Introduction:**

Linguistic choices rapidly evoke social meanings, that is, information related to social identities, group membership, and social evaluations of the speaker, the hearer, and their relationship, but this activation is influenced by the linguistic domain. Moreover, social meaning and language attitudes are shaped by the societal context, but most previous studies focused on monolingually oriented societies.

**Method:**

We report findings from the German-speaking minority in Namibia, a multilingually oriented society. We conducted an open-guise study (Soukup, 2013) that examined how linguistic choice in different linguistic domains (lexical vs. grammatical choice) evokes social meaning. Namibian German speakers evaluated the same (Namibian German) speaker producing a text in three conditions: (1) in Standard Germany German (DE-stand); (2) with some words replaced by lexical borrowings from English and Afrikaans characteristic for Namibian German (NAM-lex); and (3) with grammatical changes characteristic for Namibian German (NAM-gram).

**Results:**

DE-stand forms were associated with high competence ratings and Germany as the speaker’s place of origin and residence, but also with communicative situations in Namibia where competence in Standard German is important (e.g., at the office). In contrast, NAM-lex led to high solidarity ratings, lower competence ratings, and Namibia as the speaker’s place of origin and residence, and was associated with communicative situations in Namibia where ingroup membership is important/salient (interaction with friends). NAM-gram evoked social meaning in an interesting intermediate way: while the speaker’s place of origin tended to be associated with Namibia, their place of residence was associated with both Germany and Namibia, and participants avoided high ratings on solidarity and competence items.

**Discussion:**

We discuss the results with respect to the identity construction of Namibian German speakers, who use Namibian German linguistic characteristics to distinguish themselves from Germany German speakers (i.e., tourists) but nevertheless associate Standard Germany German with high prestige and competence.

## 1 Introduction

Questions of how social information is transmitted via language use and how people evaluate language and speakers have been investigated by researchers in psychology and linguistics (Bekker, 2019; Dragojevic et al., 2021). In psychology, *language attitudes*, i.e., evaluations of language users, are often linked to emotional, behavioural, and cognitive components (Bekker, 2019; Dragojevic et al., 2021) and are related to interlocutors’ group memberships, intergroup relations, and social identities (Tajfel and Turner, 2004; Bourhis and Maass, 2005; Bekker, 2019; Dragojevic et al., 2021). In linguistics, the term *social meaning* is used to “capture the idea that speakers and listeners use linguistic structures to carry social information and thus shape the situations and larger societal structures in which they participate” (Campbell-Kibler, 2009, p. 135), and linguistic choices are understood to index group memberships in production and comprehension (Labov, 2001; Eckert, 2008).

Research from both fields demonstrated that linguistic choices rapidly evoke social meaning, i.e., language stereotypes and language attitudes towards the speaker (Bourhis and Maass, 2005; Bekker, 2019; Dragojevic et al., 2021). Speakers of standard language varieties are usually evaluated highly on competence measures (e.g., intelligent, successful), whereas speakers of non-standard varieties are associated with less competence (e.g., Trudgill, 1986; Preston and Robinson, 2005; Bekker, 2019; Dragojevic et al., 2021). These evaluations reflect language stereotypes and ideologies, i.e., “sets of interested positions about the role and use of language that attempt to justify and rationalize linguistic diversity” (Dragojevic et al., 2013, p. 3), held in society at large (cf., Albury, 2020. Lippi-Green, 1997). In addition, language attitudes and language use are also related to group identity construction (Giles and Johnson, 1987; Tajfel and Turner, 2004). Speakers of non-standard varieties may be evaluated highly in terms of solidarity (e.g., friendliness, familiarity), especially by ingroup members (Trudgill, 1986; Preston and Robinson, 2005; Bekker, 2019; Dragojevic et al., 2021), reflecting the *covert* prestige associated with non-standard varieties in contrast to the (*overt*) prestige associated with standard varieties (cf. Labov, 1985). In production, speakers may consciously or unconsciously choose between several linguistic forms associated with different varieties (e.g., pronouncing ING as *-in* or as *-ing*) or languages to evoke different facets of social meaning and create different social personas and to promote or reject ingroup identity and/or differentiation from outgroups (Labov, 2001; Bucholtz and Hall, 2005; Eckert, 2008; Campbell-Kibler, 2009; Stell and Dragojevic, 2017).

The activation of language stereotypes was found to be influenced by several factors, e.g., task demands, ingroup-outgroup relations (e.g., Bekker, 2019; Dragojevic et al., 2021), and – particularly relevant for the present study – linguistic domain: accent and lexical choice evoke social meaning more strongly compared to syntactic patterns such as word order choices (Labov, 2001; Matras, 2009; Meyerhoff and Walker, 2013; Wiese et al., 2022; Levon and Buchstaller, 2015).

Most previous research on language attitudes or social meaning investigated the perception of language attitudes in monolingually oriented societies (but see Giles and Watson, 2013; Bekker and Levon, 2017; Stell and Dragojevic, 2017; Álvarez-Mosquera et al., 2025). In such societies, monolingualism is seen as the norm, and one language (usually the majority language) is privileged over others (e.g., Gogolin, 2008, 2021; Albury, 2020). However, this kind of societal context is mostly restricted to the Global North, while in the Global South, most societies are multilingually oriented. In such societies, multilingualism and linguistic diversity are regarded as an everyday normalcy and are often institutionally promoted (e.g., Albury, 2020; Gogolin, 2021). These differences in societal attitudes and ideologies are expected to be reflected at the individual level in multilingual practices (e.g., widespread language mixing in multilingually oriented societies) and identity construction (Lippi-Green, 1997; Rampton, 2017), and also in the evaluation of speakers and ethnolinguistic groups producing variations in linguistic forms (Dragojevic et al., 2013).

We report an open-guise study in which we investigated language attitudes in the German-speaking community in Namibia. In particular, we examined the interplay between the role of the linguistic domain, the social meaning of Namibian German in comparison to Standard Germany German, the variety of German that is considered standard language (with concomitant assumptions of “correctness”) in Germany, and the identity construction of minority speakers. Our study further extends previous work by investigating social meaning with respect to both speaker factors (evaluation, supposed place of origin, and residence) and situational factors, i.e., aspects that are usually studied separately (Stell and Dragojevic, 2017, but see Buschfeld and Schröder, 2019).

## 2 Background

### 2.1 Societal background: German-speaking minority in Namibia

Namibia is a multilingual society in sub-Saharan Africa, with English as the official language and 13 national languages, including Bantu and Khoisan languages (e.g., Oshivambo, Nama/Damara) and two Indo-European languages, Afrikaans and German. English is used in government and official business and education, whereas national languages (“mother tongues”) may be used as the language of instruction in school until grade 5 (Shah and Zappen-Thomson, 2018). In 2011, the most frequently spoken languages at home were Oshivambo (49%) and Nama/Damara languages (11%), whereas Afrikaans was used in 10%, English in 3%, and German in 1% of the households, according to the Population and Housing Census (Namibian Statistics Agency, 2011, p. 68).

The status of the Indo-European languages Afrikaans, German, and English relates to Namibia’s colonial history. Afrikaans was prevalent at the beginning of the 19th century and spread as a lingua franca around 1810–1840 (Groenewald, 2010). Namibia was a colony of the German Empire during 1884–1915, during which the German colonizers expelled the local tribes from their land, compelled them to work as paid labour on farms, and committed genocide on the Herero and Nama people (Kellermeier-Rehbein, 2016; Olagunju and Oladayo, 2025). German was the official language and taught in school for white students, but Afrikaans continued to be used (alongside German) for teaching and diplomacy and as a lingua franca for inter-ethnic communication (Gretschel, 1995; Groenewald, 2010). After World War I, Namibia was under the South-African mandate until the end of apartheid (1920–1990). Afrikaans and English became official languages, but Afrikaans continued to be dominant in public settings (Böhm, 2003; Kellermeier-Rehbein, 2016; Stell and Dragojevic, 2017).

With independence in 1990, English was chosen as the official language (Böhm, 2003; Stell and Dragojevic, 2017). It is reported to be associated with high overt prestige and high economic value (Stell and Dragojevic, 2017). In contrast, Afrikaans has a low overt prestige due to its relation to the colonial history; yet both languages are used for inter-ethnic communication (Stell and Dragojevic, 2017; Buschfeld and Schröder, 2019). German has high prestige within the German-speaking minority (Buschfeld and Schröder, 2019), but its status in the general Namibian society is less clear. While it may be less prestigious compared to English and Afrikaans (Buschfeld and Schröder, 2019), German as a foreign language has become more popular in recent years (2016: 1,329) even outnumbering the number of students studying German as mother language (2016: 1,329) (Shah and Zappen-Thomson, 2018 citing data from the *Goethe Institute Namibia* and the *Arbeitsgemeinschaft des Deutscher Schulvereine in Namibia*, two organizations that promote the acquisition and learning of German in Namibia, the former organized by Germany, the latter by the local Namibian German community).

The German-speaking minority in Namibia comprises around 20,000 speakers (1% of the population) and continues to be a privileged minority group with a high income and high levels of education (Shah and Zappen-Thomson, 2018; Riehl and Beyer, 2021). It is a vital language community that differs from other German minority settings in that the community comprises a wide age range from young to older speakers and that German is spoken not only in family contexts but also in the public domain, e.g., in shops or job settings (Wiese et al., 2014, 2017; Shah and Zappen-Thomson, 2018). Most speakers are trilingual, speaking German, English, and Afrikaans, with some speakers also having competencies in Oshivambo, Otjiherero, and Nama/Damara (Leugner, 2023), with English and Afrikaans usually being used in interethnic communication. Especially younger speakers seem to share societal language attitudes towards English and Afrikaans, considering English as more prestigious (and urban) and Afrikaans as less prestigious (and rural) (Leugner, 2023). German is usually seen as a prestigious language across generations, as it provides access to higher education and a career in Germany (e.g., Häusler, 2018; Buschfeld and Schröder, 2019).

Colloquial Namibian German is usually acquired in the family, whereas Standard German is learned via formal instruction in school (Leugner, 2023). German is used as the language of instruction, especially in private schools where German is often taught by teachers from Germany, and students have the possibility to do the German “Abitur” (final school exam) to gain access to German universities (Dück, 2018; Shah and Zappen-Thomson, 2018). Moreover, the German-speaking minority comes in contact with Standard Germany German and non-standard German varieties from Germany via the internet, social media, German TV, visits to Germany, and through interactions with tourists from Germany (Leugner, 2023; Shah and Zappen-Thomson, 2018).

### 2.2 Namibian German features and social salience

Namibian German differs from the Standard German variety spoken in Germany (DE-stand) and shows differences from but also similarities to other non-standard German varieties (Wiese and Bracke, 2021; Zimmer, 2021). Characteristics of Namibian German affect all linguistic levels and arise from different factors related to the multilingual contact situation, specifically transfer from the contact languages (Shah, 2007; Riehl, 2014), internal processes within German that are also evident in other German varieties (Wiese et al., 2014), and dialect levelling mechanisms arising from contact of different German regional varieties brought to Namibia by the first immigrant generation (Zimmer, 2021). In the following, we briefly consider features at different linguistic levels that are relevant to our empirical study.

With respect to phonetics, previous work highlighted northern-German dialect characteristics for Namibian German (Riehl, 2014; Stuhl and Zimmer, 2021). A recent corpus study found that some of these features occur frequently in elder speakers but less in younger speakers, possibly due to contact with Standard Germany German via teachers from Germany and contact with Germany-based media (Stuhl and Zimmer, 2021).

Namibian-specific features at the lexical level (NAM-lex) include borrowings of words from Afrikaans and English to fill lexical gaps (e.g., *braai*, a special type of barbecue), and English or Afrikaans equivalents for DE-stand words (*trolley/trollie* for DE-stand *Einkaufswagen*, *alright/oraait* for DE-stand *in Ordnung*, *phone* for DE-stand *Handy*). Usually, these words are morphologically integrated, as illustrated by the verbal inflection in *braaien* (“to barbecue” for DE-stand *grillen*) (Shah, 2007; Riehl, 2014; Wiese and Bracke, 2021; Zimmer, 2021).

With respect to grammatical features (NAM-gram), we find transfer as well as internal developments. As an example of the first pattern, new forms involving the choice of existing words can develop through grammatical transfer from a contact language. For instance, the NAM-gram variant *weh kriegen* (“to hurt”) differs semantically and structurally from the DE-stand form *weh tun* (“to hurt”), and instead shows parallels to Afrikaans *seer kry* (“to get pain”) (Wiese and Bracke, 2021). As a second example, the NAM-gram variant *spät sein* (“to be late”) differs from the DE-stand variant *zu spät sein* in that it works without the particle *zu* (“too”), a pattern that is parallel to Afrikaans *laat wees* and English *to be late* (Wiese and Bracke, 2021).

Word order phenomena may also result from transfer (Shah, 2007; Riehl, 2014). An example in which transfer from Afrikaans may occur is subordinate sentences with verb-second, that is, where the finite verb is in second position, that follow a matrix sentence with a negation (Shah, 2007; Zimmer, 2021), as in *Ich glaub nicht, die hat wirklich aufgepasst.* (lit. “I believe not, she has really paid attention”, “I don’t believe she was really paying attention”). The DE-stand variant requires a subordinate clause with a complementizer and the finite verb in final position, as in *Ich glaub nicht, dass die wirklich aufgepasst hat*. (lit. “I believe not, that she really attention paid has.”). A second word order phenomenon may also result from internal developments given that they also occur in non-standard German varieties in Germany (Wiese, 2011, 2013; Winkler, 2014; te Velde, 2017; Walkden, 2017; Alexiadou and Lohndal, 2018; Wiese and Müller, 2018; Bunk, 2020). In these constructions, the finite verb of a main clause is placed in the third position, as in *Auf einmal ein Auto ist da von hinten gekommen.* (lit. “suddenly a car has from behind come”; “suddenly a car came from behind”) rather than in the second position, as in the DE-stand variant *Auf einmal ist da ein Auto von hinten gekommen* (lit. “suddenly has a car come from behind”).

Finally, NAM-gram features may also be morphological, e.g., case marking information may be missing or be different from DE-stand German. In contrast to what has been reported for other contact varieties of German, this phenomenon is relatively rare in Namibian German (Zimmer, 2021).

Several studies investigating the frequency of different Namibian German features and the situational factors influencing their occurrence were based on the DNam-Korpus (Zimmer et al., 2020). The corpus documents the language use and attitudes of the German-speaking minority in Namibia. It comprises free conversations, semi-structured interviews, and elicited production data from 110 speakers, aged 6–75 years. The elicited production data used the *Language Situations Methods* (Wiese, 2020), in which speakers play-act, in a simulated conversation, telling different addresses about an event they witnessed. For the DNam data, the event was a traffic accident, and speakers play-acted telling a German teacher from school (a role taken by the elicitor) and a close person (a friend or family member) about it. Several studies (Wiese and Bracke, 2021; Wiese et al., 2022; Sauermann et al., 2023; Schulte, 2025) found that NAM-lex features were almost exclusively used when addressing a friend or family member rather than a teacher. NAM-gram features (e.g., choice of existing words, word order) were produced more frequently when addressing a friend compared to a teacher. However, they tended to occur more often in speech addressed to the teacher compared to NAM-lex features (Wiese and Bracke, 2021; Wiese et al., 2022; Sauermann et al., 2023; Schulte, 2025).

**Figure 1:**
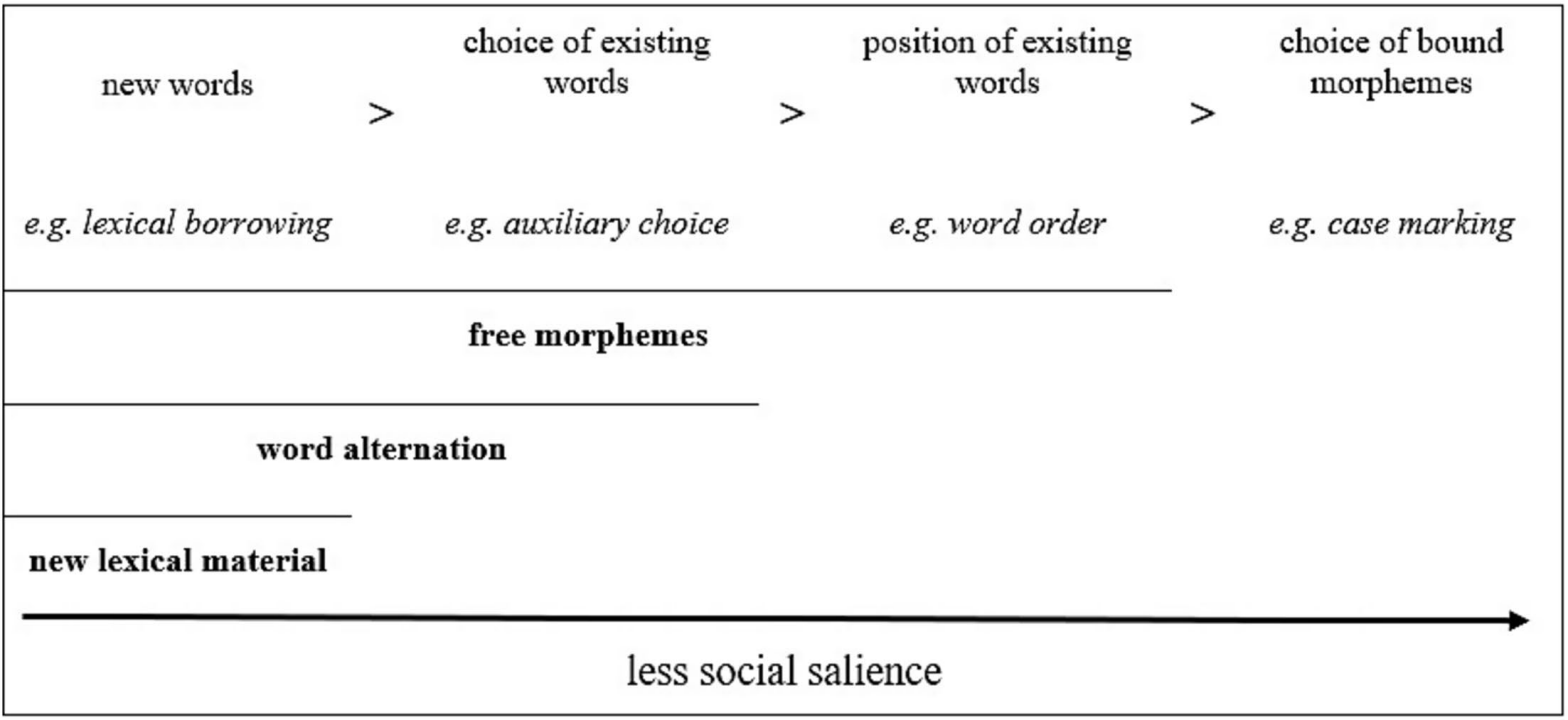
Social saliency hierarchy (reprint from Figure 16.3, Wiese et al., 2022, p. 315).

Along with evidence from an experimental study, Wiese et al. (2022) explain the differences between NAM-lex and NAM-gram features in terms of a Social Saliency Hierarchy (see Figure 1), arguing that linguistic features differ in their social salience, i.e., their “relative ability […] to evoke social meaning (Levon and Fox, 2014, S. 185), depending on how much overt and novel material they involve. According to this hierarchy, NAM-lex features (e.g., lexical borrowings) have the highest social salience because they involve new words, followed by NAM-gram features that affect the choice of existing words (e.g., auxiliary choice) and then those that only affect the position of existing words (e.g., word order variation), and finally those that only affect the choice of bound morphemes (e.g., case marking variation).

### 2.3 Present study

The present study was conducted together with a study by Schulte et al. (under review) that examined the impact of the linguistic features of Namibian-specific features on the addressee identification. In the study by Schulte et al. (under review), Namibian German speakers listened to audio recordings in Standard Germany German (DE-stand), German with Namibian-specific lexical features (NAM-lex), and German with Namibian-specific grammatical features (NAM-gram) and indicated whether the recordings were addressed to a friend or a teacher. The results indicate a clear impact of the linguistic forms on the imagined addressee. DE-stand was associated with speaking to a teacher, whereas the use of NAM-lex was associated with speaking to a friend. Again, NAM-gram features revealed an interesting intermediate pattern, being associated both with speaking to a teacher and to a friend.

Drawing on the same materials, we investigated how the different linguistic forms influence the perception of the speaker. In particular, we ask:

1) What social meaning does the Namibian German community in Namibia associate with Standard Germany German (DE-stand) and with Namibian German patterns (NAM-lex and NAM-gram)?

Social meaning will be captured by ratings of the speakers with respect to competence and solidarity measures, assumptions about speaker’s place of origin and residence, and about possible situations where one might meet the speaker. DE-stand is expected to be associated with high competence (Preston and Robinson, 2005; Bekker, 2019; Dragojevic et al., 2021), Germany as a place of origin and residence, and the use of DE-stand in interactions with Germans or in educational settings (Leugner, 2023; Schulte et al., under review). Namibian German was expected to be related to lower competence, but higher solidarity, with Namibia as the place of living and residence, and with usage in private settings.

2) How does the linguistic domain (NAM-lex vs. NAM-gram) influence the potential to evoke social meaning?

As NAM-lex features are predicted to have a higher potential to evoke social meaning than NAM-gram features (Wiese et al., 2022; Labov, 2001; Meyerhoff and Walker, 2013), we expect higher ratings for solidarity measures and clearer patterns with respect to place of origin and residence for NAM-lex features in comparison to NAM-gram features.

3) How are identity constructions related to language attitudes, i.e., identity-related evaluations such as solidarity and place of origin and residence?

The use of Namibian German features, especially NAM-lex features, is expected to result in high solidarity ratings and, in association with Namibia as place of origin and residence, as these features index belonging to the German-speaking minority in Namibia and its distinction from German tourists (Wiese et al., 2022; Leugner, 2023).

## 3 Materials and methods

### 3.1 Participants

A total 35 speakers of the German-speaking minority in Namibia (28 participants aged between 19–38, seven participants above 40 years) participated in the study. They were recruited via existing social networks in the minority, as well as the snowball system. Participants indicated that they grew up in Namibia and use German on a regular basis in their daily lives.

### 3.2 Materials

#### 3.2.1 Sound files

The same audio stimuli as in the study by Schulte et al. (under review) were used. Three versions of a text (167–169 words) with descriptions of an accident were created: (1) a version in Standard Germany German (DE-stand), (2) a version in which four words were replaced by lexical borrowings used in Namibian German (*Trolley/Trollie* “shopping trolley”, *Phone*, *paniken* “to panic”, *oreit/alright* “alright”) (NAM-lex), and (3) a version with grammatical patterns used in Namibian German (*spät sein* “to be late”, *weh kriegen* “to hurt”), matrix clauses with verb-second after negation, and verb-third structures (NAM-gram) (see Section 2.2.). Table 1 lists the items in the three conditions. The full transcripts of the texts are provided in the Supplementary materials.

**Table 1:** Experimental items used.

Standard German (DE-stand)	NAM-lex	NAM-gram
*[…] die is da mit ihrm Einkaufswagn lang gelaufn* ‘.she went there with her shopping trolley.’	*[…] die is da mit ihrm Trolley lang gelaufn* ‘… she went there with her shopping trolley.’	*[…] die is da mit ihrm Einkaufswagn lang gelaufn* ‘… she went there with her shopping trolley.’
*Die war an ihrem Handy.* ‘She was on her mobile phone.”	*Die war an ihrem Phone.* ‘She was on her mobile phone.’	*Die war an ihrem Handy.* ‘She was on her mobile phone.’
*[….] aber mit ihr war alles in Ordnung.* ‘…but she was ok.’	*[…] aber mit ihr war alles oreit/alright.* ‘…but she was ok’	*[…] aber mit ihr war alles in Ordnung* ‘…but she was ok.’
*Da ist die hingefalln und hat sich erschrocken.* ‘She fell down and was frightened.’	*Da ist die hingefalln und hat gepanikt.* ‘She fell down and was frightened.’	*Da ist die hingefalln und hat sich erschrocken.* ‘She fell down and was frightened.’
Ähm, tut mir leid, dass ich zu spät bin. ‘Um, sorry I’m late.’	*Ähm, tut mir leid, dass ich zu spät bin* ‘Um, sorry I’m late.’	*Ähm, tut mir leid, dass ich _ spät bin.* ‘Um, sorry I’m late.’
*[…] die Frau hat sich zum Glück nicht doll wehgetan.* ‘Luckily, the woman did not hurt herself badly.’	*[…] die Frau hat sich zum Glück nicht doll wehgetan.* ‘Luckily, the woman did not hurt herself badly.’	*[…] die Frau hat sich zum Glück nicht doll weh gekriegt.* ‘Luckily, the woman did not hurt herself badly.’
*Ich glaub nicht, dass die wirklich aufgepasst hat.* ‘I do not believe that she really paid attention.’	*Ich glaub nicht, dass die wirklich aufgepasst hat.* ‘I do not believe that she really paid attention.’	*Ich glaub nicht, die hat wirklich aufgepasst.* ‘I do not believe that she really paid attention.’
*Und auf einmal ist da ein Auto von hinten gekommen.* ‘And suddenly a car came from behind.’	*Und auf einmal ist da ein Auto von hinten gekommen.* ‘And suddenly a car came from behind.’	*Und auf einmal ein Auto ist da von hinten gekommen.* ‘And suddenly a car came from behind.’

All three versions of the text spoken by two Namibian German speakers, one female, one male, both aged 24 years, who grew up in Namibia. To ensure the comparability of the three versions, for each speaker, one version of the recordings was taken as a base, with the replacements (taken from the other versions of the same speaker) being inserted into the base version.

#### 3.2.2 Questionnaire

The questionnaire consisted of three parts. The first part asked open questions about each speaker: estimated age, place of origin, place of residence, and where one might meet them. Responses about speakers’ ages are not reported here because they were not influenced by the experimental condition, and the majority of participants estimated the age of the speakers between 18–25 years. The second part of the questionnaire contained semantic differentials for dimensions of competence (*kompetent* “competent”, *ehrgeizig* “ambitious”, *intelligent* “intelligent”*, beruflich erfolgreich* “successful”, *selbstbewusst* “self-confident”) and solidarity (*entspannt* “relaxed”, *humorvoll* “humorous”, *sympathisch* “likeable”, *vertraut* “familiar”, *freundlich* “friendly”) that were arranged on a 7-point Likert scale ranging from no agreement with the characteristics (e.g., *überhaupt nicht kompentent* “not competent at all”) to full agreement (e.g., *sehr kompetent* “very competent”). Characteristics were arranged in such a way that no more than two characteristics of each dimension (competence vs. solidarity) were presented directly after each other. The third part of the questionnaire consisted of an open question that invited participants to mention further characteristics of the speaker (*Fallen dir spontan noch weitere Eigenschaften zu dieser Person ein?* “Do additional characteristics of the speaker come to your mind spontaneously?”).

### 3.3 Design and procedure

The study was conducted as an open-guise study (Soukup, 2013), an extension of the matched-guise method (Lambert et al., 1960), in which participants were explicitly informed that they would listen to texts produced by the same speaker.

The study was conducted via the online platform IbexFarm (Zehr and Schwarz, 2018). The auditory stimuli were presented as mock audio messages received on a phone. All participants listened to all three recordings of one speaker (either the male or the female speaker), with the order of presentation of the recordings being counterbalanced among participants. After each presentation of the stimulus, participants were asked to fill in the questionnaire, i.e., to evaluate the speaker. At the end of the study, participants provided their demographic information (e.g., their age, place of living, and language background).

## 4 Results

### 4.1 Quantitative results

#### 4.1.1 Speaker evaluation

The evaluations of the speakers were analyzed separately using cumulative linear mixed effects models (clmm), which are suitable for ordinal data elicited by Likert scales (Veríssimo, 2021). Subsequently, a factor analysis was calculated (i) to test whether the ratings support the expected differentiation along the two dimensions (competence and solidarity) and (ii) to summarize the ratings on each dimension and investigate the relationship between the evaluations and the social meaning aspects related to expected place of origin and residence and location of encounter.

Clmms were calculated in the R Studio environment (R Core Team, 2025) using the *ordinal* package (Christensen, 2025). The models estimated the fixed effects of Condition, Gender, and their interaction, and the random effects of Participants on the evaluation regarding each semantic differential. The contrast coding of Condition and Gender were treatment contrasts, with Standard Germany German (DE-stand) and the female speaker as the baseline. Model fitting was performed in a stepwise fashion, starting with the most complex model that included the full factorial set of random effects (random slope-adjustment for all fixed effects and interactions for Participants). Due to convergence errors of the maximal models, the complex models were trimmed down in a stepwise fashion using log-likelihood tests for model comparisons (Baayen, 2008; Bates et al., 2018). The procedure always resulted in the choice of the simplest model that included only intercept adjustments. Moreover, the fitting procedure always identified models as optimal in which the effects of Gender and the interaction between Gender and Condition were removed.

**Figure 2:**
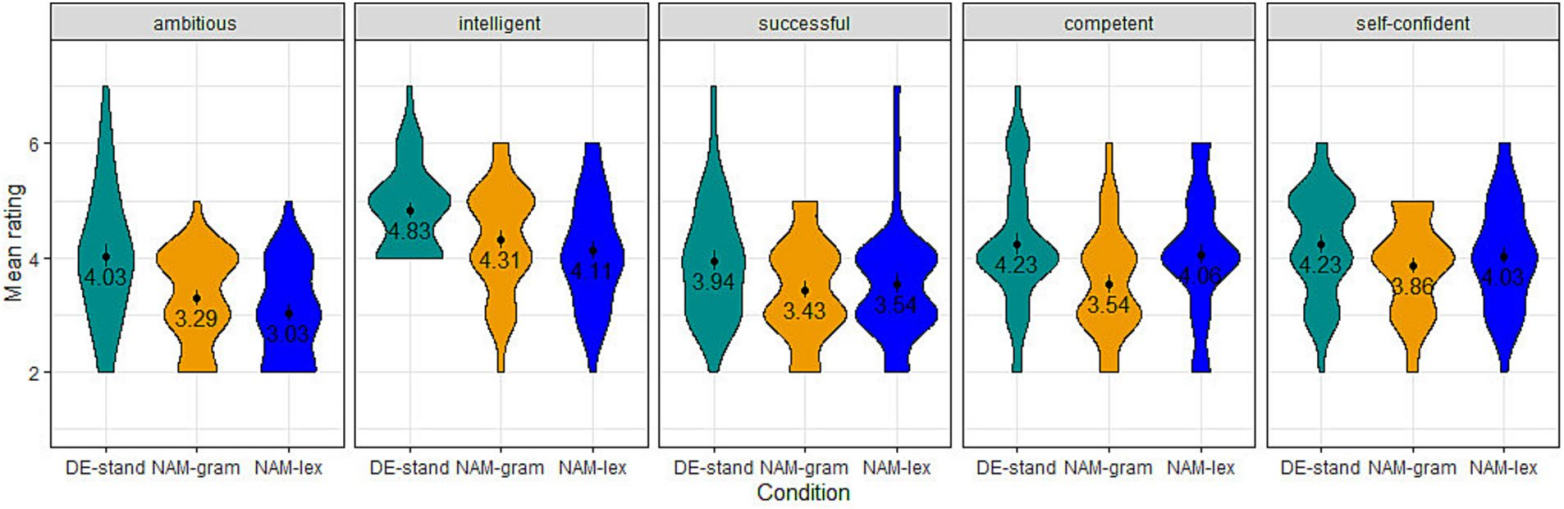
Mean ratings on the competence dimension separated by condition.

Figure 2 shows the mean ratings separated by Condition on the competence dimension. The ratings for the differentials, *ambitious*, *successful*, and *intelligent* patterns, together. Speakers received higher ratings for these characteristics in the DE-stand condition than in the NAM-lex (ambitious: *b* = −3.136, *SE* = 0.634, *z* = −4.949, *p* < 0.001; intelligent: *b* = −1.761, SE = 0.501, *z* = −3.515, *p* < 0.001; successful: *b* = −1.182, *SE* = 0.488, *z* = −2.425, *p* = 0.015) and in the NAM-gram condition (ambitious: *b* = −2.262, *SE* = 0.574, *z* = −3.943, *p* < 0.001; intelligent: *b* = −1.200, *SE* = 0.475, *z* = −2.526, *p* = 0.0115; successful: *b* = −1.206, *SE* = 0.486, *z* = −2.479, *p* = 0.013). Ratings for the differentials *competent* and *confident* were higher in the DE-stand condition than in the NAM-gram condition (competent: *b* = −1.745, *SE* = 0.506, *z* = −3.446, *p* < 0.001; confident: *b* = −0.986, *SE* = 0.481, *z* = −2.051, *p* = 0.040), but did not differ from the NAM-lex condition (competent: b = −0.490, SE = 0.480, *z* = −1.020, *p* = 0.308; confident: *b* = −0.523, SE = 0.469, *z* = −1.113, *p* = 0.266), but *competent* ratings were higher in the NAM-lex than in the NAM-gram condition (*b* = 1.255, *SE* = 0.487, *z* = 2.580, *p* = 0.01).

**Figure 3:**
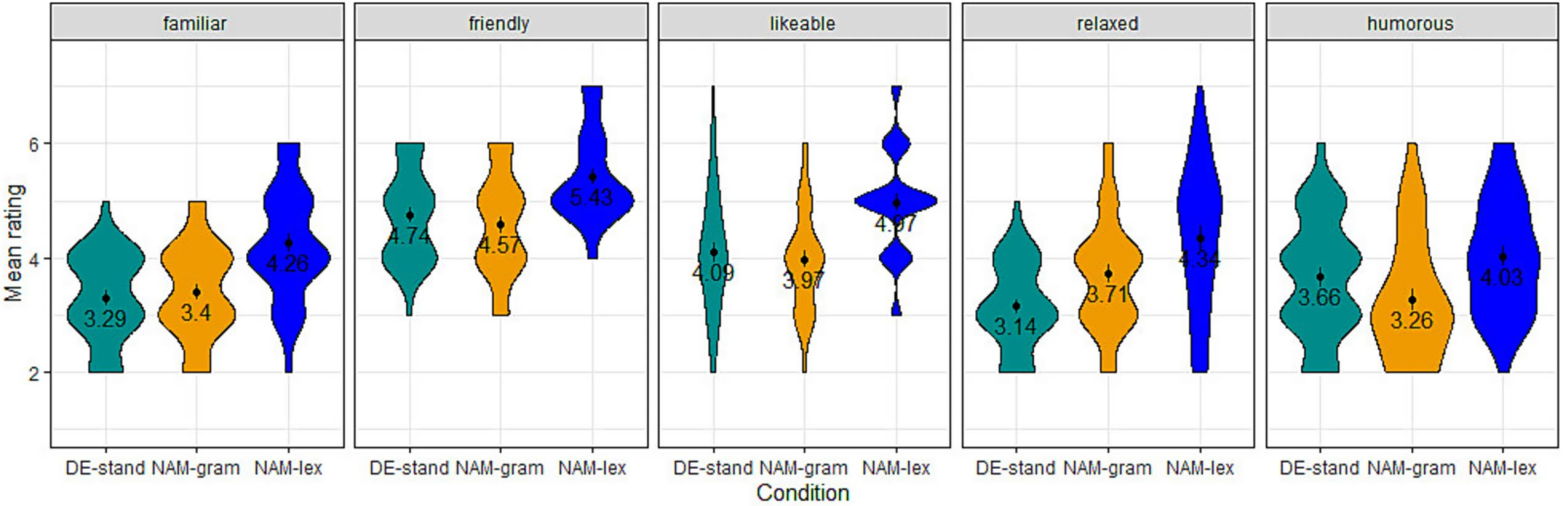
Mean ratings on the solidarity dimension separated by condition.

Figure 3 shows the mean ratings separated by Condition on the solidarity dimension. The ratings for *familiar*, *friendly*, and *likeable* patterns together. The ratings for these characteristics were lower in the DE-stand condition than in the NAM-lex condition (familiar: *b* = 2.294, *SE* = 0.530, *z* = 4.325, *p* < 0.001; friendly: *b* = 2.305, *SE* = 0.555, *z* = 4.153, *p* < 0.001; likeable: *b* = 2.286, *SE* = 0.535, *z* = 4.275, *p* < 0.001), but did not differ from the NAM-gram condition (familiar: *b* = 0.267, *SE* = 0.4558, *z* = 0.585, *p* = 0.558; friendly: *b* = −0.6456, *SE* = 0.501, *z* = −1.288, *p* = 0.198; likeable: *b* = −0.2093, SE = 0.4657, *z* = −0.449, *p* = 0.653). Ratings for the differential *relaxed* were lower in the NAM-gram than in the NAM-lex condition (*b* = 1.382, *SE* = 0.486, *z* = 2.844, *p* < 0.01) but higher than in the DE-stand condition (*b* = −1.345, *SE* = 0.474, *z* = −2.836, *p* < 0.01). *Humorous* ratings were lower in the NAM-gram than in the NAM-lex condition (*b* = 1.675, *SE* = 0.487, *z* = 3.442, *p* < 0.001), and marginally lower than in the DE-stand condition (*b* = 0.914, SE = 0.468, *z* = 1.955, *p* = 0.051).

The rating results suggest that, in general, DE-stand was associated with higher competence and lower solidarity, NAM-lex with higher solidarity and lower competence, and NAM-gram with lower competence and solidarity. In addition, the evaluations associated with the competence and solidarity dimension pattern together. This is assessed by a factor analysis that aimed (1) to identify how well the evaluations load on different factors and (2) to combine the ratings on each dimension. The factor analysis was conducted using the procedure suggested by Field et al. (2012), using the *psych* package (Revelle, 2025). In order to determine the suitability of the dataset for the principal component analysis, the Kaiser-Meyer-Olkin (KMO) criterion was calculated. All variables yielded values higher than 0.5 (which is the advised cut-off point) (Field et al., 2012). The procedure led to the identification of three factor loadings (see Table 2). Four solidarity variables (*likable, friendly, relaxed, familiar*) load on Factor 1, whereas three competence variables (*competent, self-confident, successfu*l) load on Factor 2. Three items load less clearly on the first two factors: The *intelligent* item loads on a separate Factor 3. The *humorous* item loads equally well on Factors 1 and 2. The *ambitious* item also clusters on Factor 2, but also (though less strongly) on Factor 3 and negatively on Factor 1.

**Table 2:** Standardized loadings (above |0.3|) (pattern matrix) based upon correlation matrix.

Item	Factor 1	Factor 2	Factor 3
Likeable	**0.76**		
Friendly	**0.70**		
Relaxed	**0.69**		
Familiar	**0.65**		
Competent		**0.73**	
Self-confident		**0.72**	−0.36
Successful		**0.68**	
Humorous	0.44	0.47	
Ambitious	−0.35	**0.45**	0.34
Intelligent			0.89
Cumulative proportion	0.41	0.79	1.00

Bold values indicate items that strongly load on one of the two factors (Competence and Solidarity) that are considered in the later analyses.

**Figure 4:**
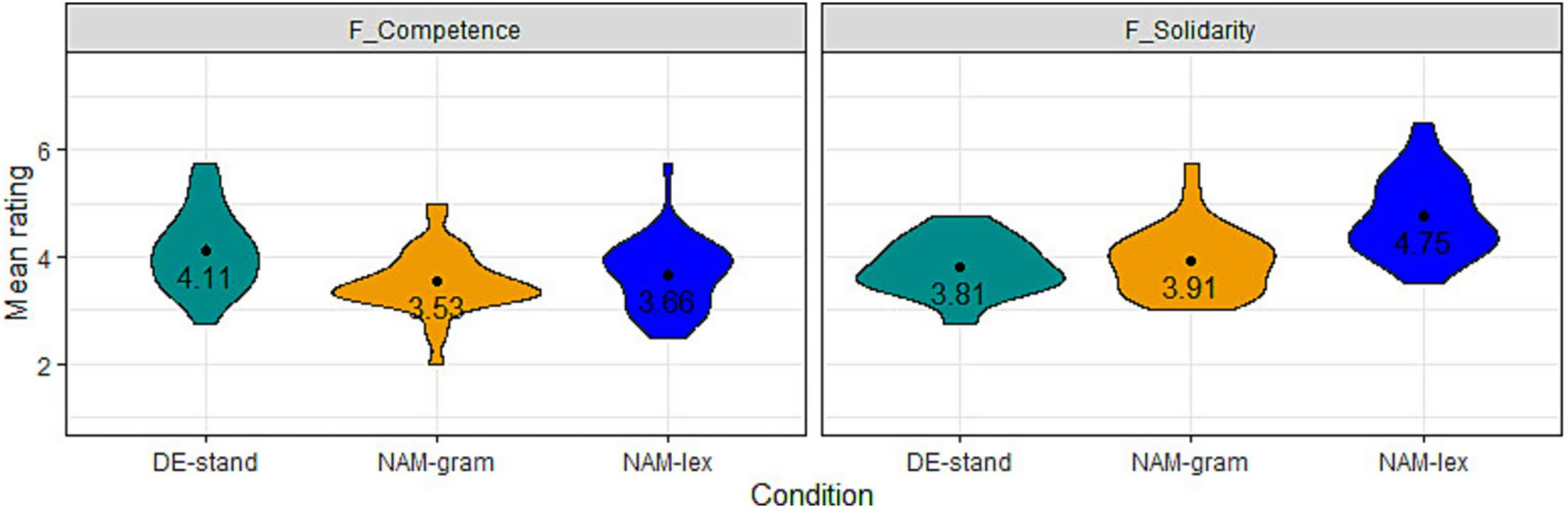
Mean ratings on the combined competence and solidarity measure separated by condition.

For subsequent analyses, the ratings for *likable, friendly, relaxed,* and *familiar* are combined to represent the *solidarity* ratings, while the ratings for *competent, self-confident, successful,* and *ambitious* are combined to represent the *competence* ratings. The combined mean ratings are shown in Figure 4 and mirror the general pattern of Figures 2, 3. Competence ratings are higher in the DE-stand than in the NAM-lex (*b* = −0.44, *SE* = 0.11, *t* = −3.900, *p* < 0.001) and NAM-gram condition (*b* = −0.58, *SE* = 0.11, *t* = −5.096, *p* < 0.001). Solidarity ratings are lower in the DE-stand than NAM-lex condition (*b* = 0.94, *SE* = 0.12, *t* = 8.002, *p* < 0.001) but did not differ from the NAM-gram condition (*b* = 0.10, *SE* = 0.12, *t* = 0.855, *p* = 0.395).

#### 4.1.2 Place of residence and origin

Answers concerning the Place of Residence and Origin were coded and summarized in three categories: Germany-related answers, Namibia-related answers, and “other” answers. The latter category included answers not related to a country (e.g., *country site, city, Europe*), which mentioned more than one country (e.g., “Namibia or South Africa”) or included indications of uncertainty (e.g., “Namibia?,” “rather Germany than Namibia”).

**Figure 5:**
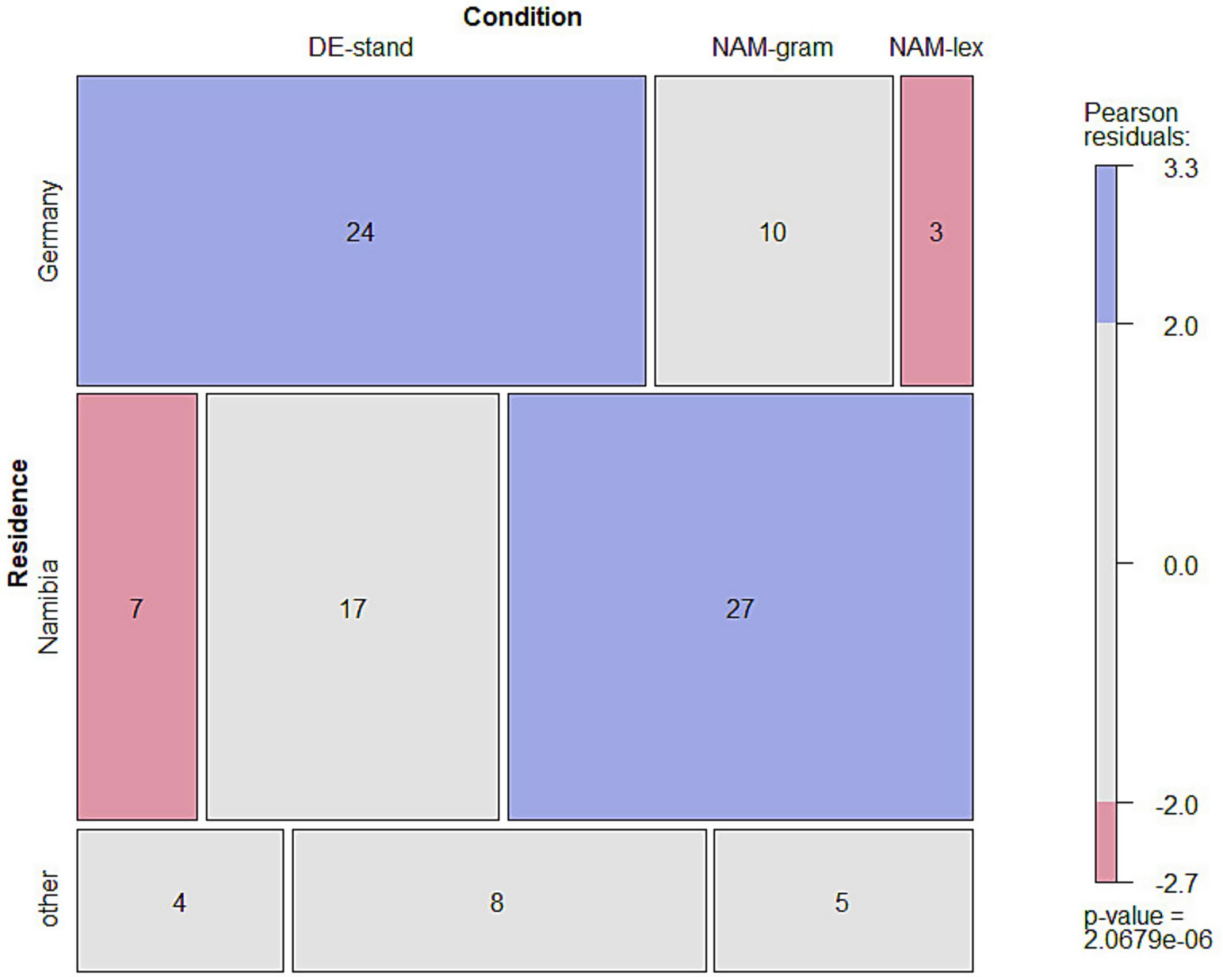
Mosaic plot for the relationship between condition and place of residence (cell numbers indicate absolute frequencies).

A chi-square test confirmed that Place of Residence and Condition interacted (χ^2^(4) = 31.835, *p* < 0.001). Figure 5 shows the residual plots for Place of Residence, created by the *mosaic* function of the *vcd* package (Meyer et al., 2024; Meyer et al., 2006; Zeileis et al., 2007). Numbers in the cells represent the observations in each condition. Red cells represent numbers of observations which are significantly lower than the expected observations, blue cells represent observations that are significantly higher than expected. The results indicate that “Germany” as a place of residence was mentioned more frequently than expected in the DE-stand condition and less frequently in the NAM-lex condition, whereas “Namibia” was mentioned more frequently in the NAM-lex condition than in the DE-stand condition. The NAM-gram condition was not associated with a particular place of residence. Moreover, responses falling into the category “other” did not vary between the conditions.

**Figure 6:**
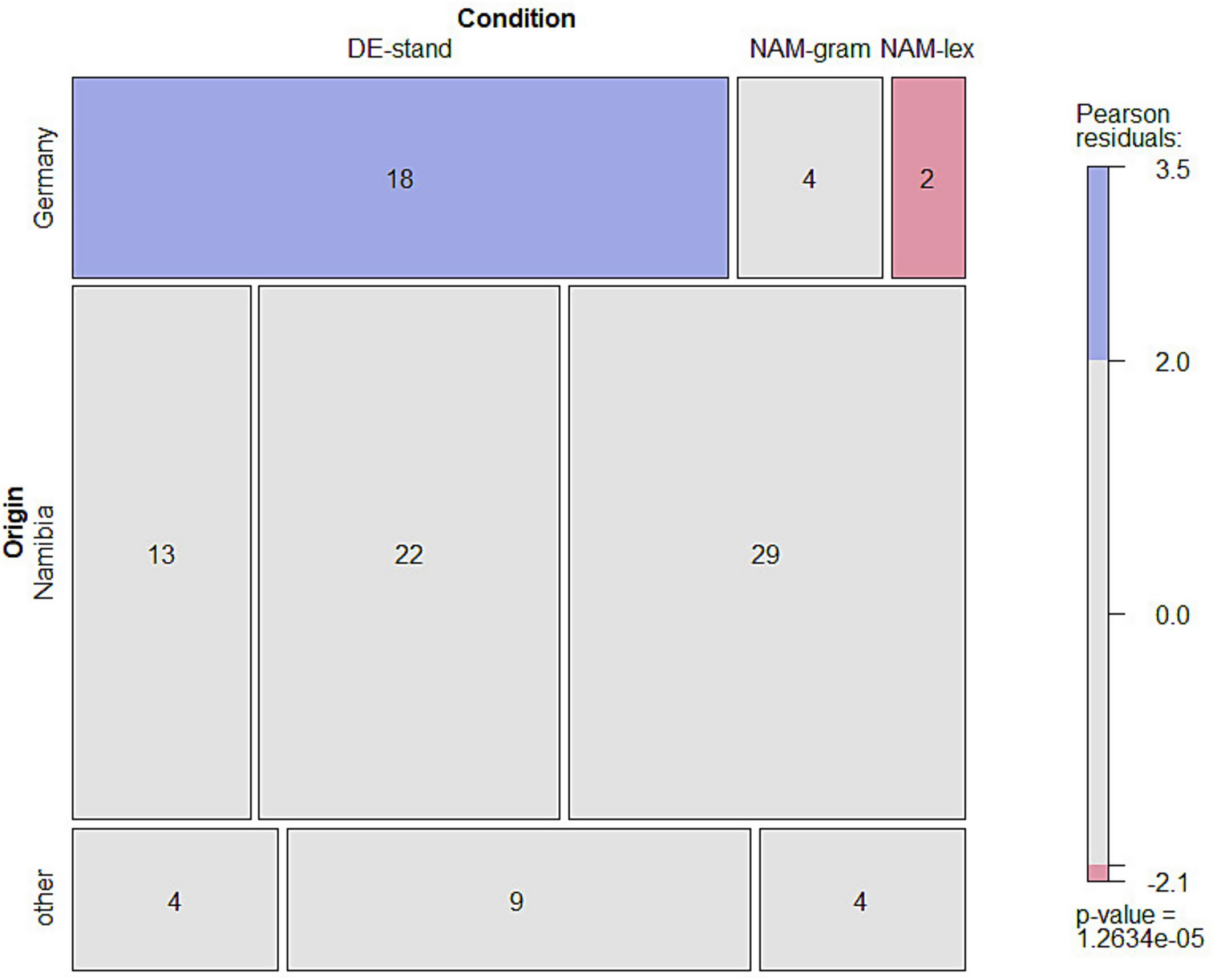
Mosaic plot for the relationship between condition and place of origin (cell numbers indicate absolute frequencies).

An additional chi-square test confirmed that Place of Origin and Condition also interacted (χ^2^(4) = 27.972, *p* < 0.001). Figure 6 shows the corresponding residual plot for Place of Origin. Only “Germany” as a place of origin was influenced by the different conditions: it was mentioned more frequently than expected in the DE-stand condition (blue cell) and less frequently than expected in the NAM-lex condition (red cell). The other two response categories (“Namibia,” “other”) were not significantly influenced by Condition (grey cells). Note that, while the frequency of “Namibia” seems to be more equally distributed across the three experimental conditions, it still seemed to be numerically somewhat more related to the NAM-lex and NAM-gram condition in comparison to the DE-stand condition.

#### 4.1.3 Relation between evaluations and place of residence/origin

Table 3 shows the relationship between the expected Place of Residence and evaluations with respect to the combined Competence and Solidarity measures. In general, higher competence ratings seem to be associated with Germany as Place of Residence and the DE-stand condition, whereas higher Solidarity ratings seem to be associated with Namibia as Place of Residence and the NAM-lex condition.

**Table 3:** Competence and solidarity ratings separated by place of residence and condition.

	DE-stand	NAM-gram	NAM-lex	Mean rating
Competence				
Germany	**4.20** (*N* = 24)	3.52 (*N* = 10)	**4.58** (*N* = 3)	4.05 (*N* = 37)
Namibia	**4.07** (*N* = 7)	3.60 (*N* = 17)	3.56 (*N* = 27)	3.64 (*N* = 51)
Other	3.62 (*N* = 4)	3.38 (*N* = 8)	3.70 (*N* = 5)	3.53 (*N* = 17)
Sum	4.11 (*N* = 35)	3.53 (*N* = 35)	3.66 (*N* = 35)	
Solidarity				
Germany	3.73 (*N* = 24)	3.78 (*N* = 10)	4.33 (*N* = 3)	3.79 (*N* = 37)
Namibia	3.86 (*N* = 7)	3.93 (*N* = 17)	**4.75** (*N* = 27)	4.35 (*N* = 51)
Other	4.25 (*N* = 4)	4.06 (*N* = 8)	5.00 (*N* = 5)	4.38 (*N* = 17)
Sum	3.81 (*N* = 35)	3.91 (*N* = 35)	4.75 (*N* = 35)	

Bold values indicate strong associations between Place of of Residence and Competence and Solidarity respectively.

Linear mixed effects models were calculated to assess the impact of the fixed effects of Place of Residence and Condition and their interaction (as well as the random effect of Participants) on the (combined) *Competence* and *Solidarity* measures, respectively. Treatment contrasts were chosen for both fixed effects with DE-stand and Germany as the baseline. The models were calculated using the *lme4* (Bates et al., 2018; Bates D. et al., 2015) and *lmerTest* package (Kuznetsova et al., 2017). The random effects structure of the models was trimmed down in a stepwise fashion, with the final models containing intercept adjustment only. For post-hoc tests, *p*-values below 0.025 were taken to be significant (Bonferroni correction, cf. Baayen, 2008). Due to low numbers of observations in the conditions, we ignore the “other” responses for the Place of Residence and Origin. With respect to *Competence*, the models revealed an interaction between Residence and Condition. If Germany was chosen as the residence, *Competence* ratings were significantly higher in the DE-stand than in the NAM-gram condition (*b* = −0.685, *SE* = 0.193, *t* = −3.548, *p* < 0.001), but did not differ from the NAM-lex condition (*b* = 0.178, *SE* = 0.327, *t* = 0.543, *p* = 0.5887). If Namibia (rather than Germany) was chosen as Residence, there was a similar difference between DE-stand and the NAM-gram condition, but *Competence* was marginally lower in the NAM-lex condition (*b* = −0.735, *SE* = 0.409, *t* = −1.797, *p* = 0.076). Post-hoc tests on Namibia responses confirmed higher *Competence* in the DE-stand than in the NAM-gram (*b* = −0.536, *SE* = 0.214, *t* = −2.509, *p* = 0.018) and NAM-lex condition (*b* = −0.572, *SE* = 0.205, *t* = −2.797, *p* < 0.009). With respect to *Solidarity*, the models did not reveal an effect of Condition if Germany was chosen as Residence. If Namibia was chosen as the residence, *Solidarity* ratings were higher in the NAM-lex than in the DE-stand condition (post-hoc: *b* = 1.055, *SE* = 0.1985, *t* = 5.311, *p* < 0.001), while the ratings in the DE-stand and NAM-gram conditions did not differ (*b* = 0.164, *SE* = 0.207, *t* = 0.790, *p* = 0.436).

Table 4 shows the relationship between expected Place of Origin and evaluations with respect to the combined *Competence* and *Solidarity* measures. In general, the pattern is similar to the results for Place of Residence. With respect to *Competence*, the models revealed an interaction between Origin and Condition. If Germany was chosen as Origin, *Competence* ratings for DE-stand were marginally higher than in the NAM-gram condition (*b* = −0.538, *SE* = 0.275, *t* = −1.955, *p* = 0.0544), while they did not differ from ratings in the NAM-lex condition (*b* = 0.518, *SE* = 0.375, *t* = 1.382, *p* = 0.1711). Note, however, that Germany as Place of Origin was chosen quite rarely in the NAM-lex (*N* = 2) and NAM-gram conditions (*N* = 4). If Namibia, compared to Germany, was chosen, NAM-lex Competence ratings were lower (*b* = −0.937, *SE* = 0.411, *t* = −2.281, *p* = 0.026). Post-hoc models for Namibia responses confirmed that *Competence* was higher in the DE-stand condition than in both the NAM-gram (*b* = −0.533, *SE* = 0.151, *t* = −3.523, *p* = 0.001) and NAM-lex conditions (b = −0.429, SE = 0.144, t = −2.970, *p* = 0.005). With respect to *Solidarity*, the model again did not reveal an effect of Condition if Germany was chosen as the Place of Origin. If Namibia was chosen, post-hoc comparisons show that *Solidarity* ratings were higher in the NAM-lex than the DE-stand condition (*b* = 1.097, *SE* = 0.159, *t* = 6.899, *p* < 0.001), while the NAM-gram condition did not differ from the DE-stand condition (*b* = 0.276, *SE* = 0.167, *t* = 1.655, *p* = 0.107).

**Table 4:** Competence and solidarity ratings separated by place of origin and condition.

	DE-Stand	NAM-gram	NAM-lex	Mean rating
Competence				
Germany	**4.26** (*N* = 18)	3.50 (*N* = 4)	**4.88** (*N* = 2)	4.19 (*N* = 24)
Namibia	**3.98** (*N* = 13)	3.56 (*N* = 22)	3.57 (*N* = 29)	3.65 (*N* = 64)
Other	3.81 (*N* = 4)	3.46 (*N* = 9)	3.75 (*N* = 4)	3.62 (*N* = 17)
Sum	4.11 (*N* = 35)	3.53 (*N* = 35)	3.66 (*N* = 35)	
Solidarity				
Germany	3.83 (*N* = 18)	3.62 (*N* = 4)	3.62 (*N* = 2)	3.78 (*N* = 24)
Namibia	3.65 (*N* = 13)	4.03 (*N* = 22)	**4.78** (*N* = 29)	4.29 (*N* = 64)
Other	4.25 (*N* = 4)	3.75 (*N* = 9)	5.12 (*N* = 4)	4.19 (*N* = 17)
Sum	3.81 (*N* = 35)	3.91 (*N* = 35)	4.75 (*N* = 35)	

Bold values indicate strong associations between Place of Origins and Competence and Solidarity respectively.

#### 4.1.4 Situation

**Figure 7:**
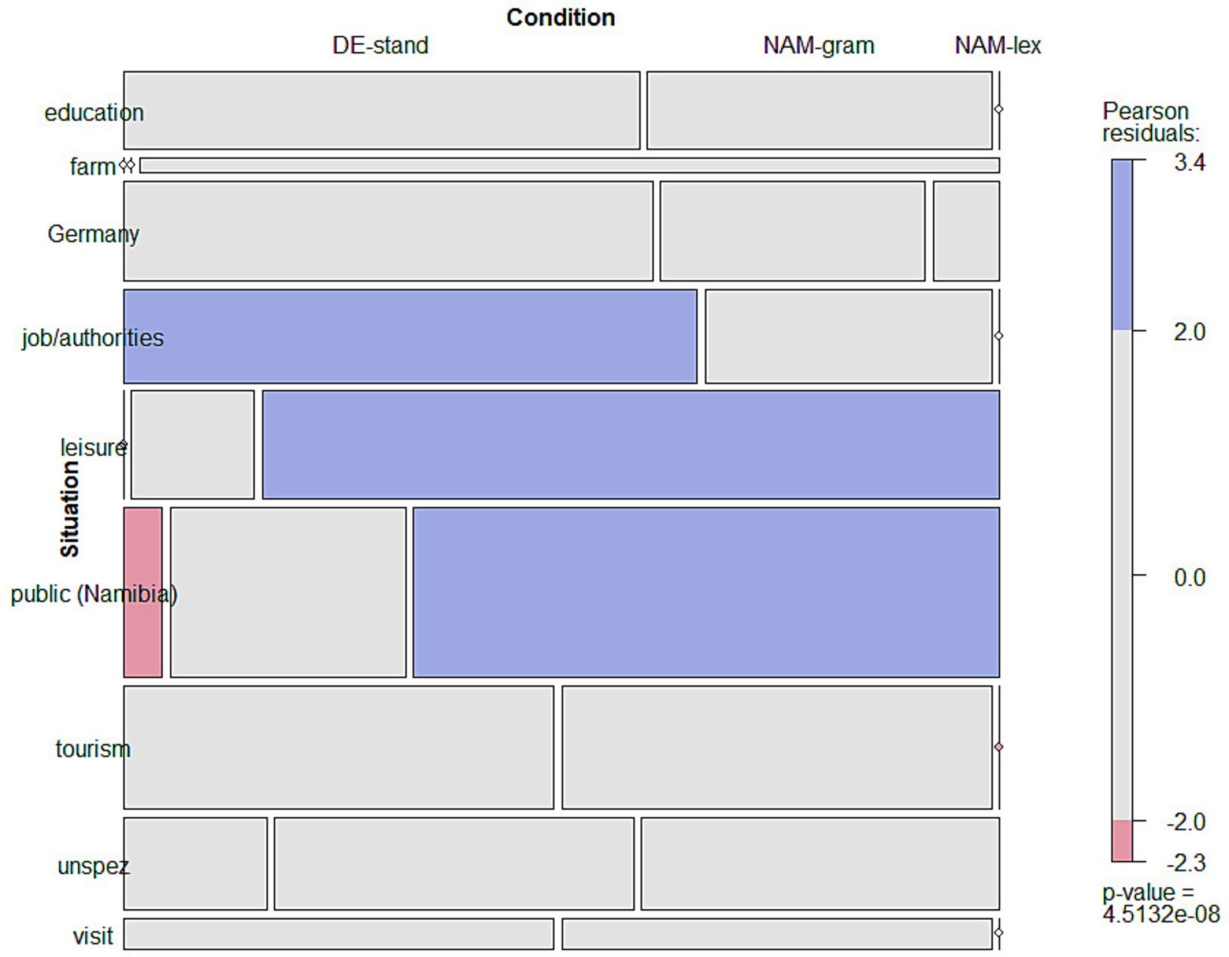
Mosaic plot for the relationship between condition and situation.

Responses about the Situation or place where one might meet the speaker were coded into 9 categories. Table 5 lists the frequency of the categories in the three conditions. A chi-square test confirmed that Situation and Condition interacted (χ^2^(16) = 66.234, *p* < 0.001). Bold numbers indicate values that are below or higher than the expected values as identified by a residual plot (see Figure 7). The results indicate that DE-stand was strongly associated with situations related to work and authorities, and less with leisure time and public space in Namibia. In contrast, NAM-lex was strongly associated with leisure time and public space in Namibia but not with interactions with tourists and authorities or at work.

**Table 5:** Competence and solidarity ratings separated by situation and condition.

	DE-stand	NAM-gram	NAM-lex
Education	6	4	0
Farm	0	0	2
Germany	8	4	1
Job/authorities	**8**	4	**0**
Leisure time	**0**	2	**12**
Public (Namibia)	**1**	6	**15**
Tourism	8	8	**0**
Visits	2	2	0
Unspecific	2	5	5

Bold values indicate responses that occur significantly above or below the expected frequency as indicated by the mosaic plot in Figure 7.

### 4.2 Qualitative results (additional characteristics)

15–17 participants added additional characteristics of the speaker in the conditions. Additions related to the DE-stand condition were related to a German tourist (English translations: “tourist”, “has sunburned skin”), to the way of speaking in Germany (“people in Kiel speak like that”, “tries to speak like in Germany”), characteristics related to competence (“business”, “young professional”, “sounds well-read”, “tries to sound authoritative”), but also more general responses (“attentive young man”, “nervous young women”, “decent because s/he calls the emergency services”).

Responses in the NAM-lex condition related to relaxed situations with friends (“relaxed dude”, “chill”, “definitely makes sundowner”, “could be a friend/mate”), speech of younger Namibians (“uses young people’s language”, “Namlish” [term for the Namibian German youth speech], “likes EES” [a popular Namibian German musician]), but also responses related to language ideology (“no appreciation of the language”), and additional characteristics (“young woman sounds self-confident and can assert herself”, “sounds excited, not relaxed”).

Responses in the NAM-gram related to relaxed situations involving tourists (“German tour guide in lodge”, “German”, “is relaxed on holiday”), to feelings of insecurity (“nervous”, “insecure”), and to attempts to speak Standard German, to sound educated, or to attempts to speak Namibian German. The responses concerning attempts to adapt to Standard German and Namibian German were numerically more frequent (seven responses) than in the NAM-lex or DE-stand condition (one response, respectively).

## 5 Discussion

Our study investigated three research questions: RQ1) What social meaning does the Namibian German community in Namibia associate with Standard Germany German (DE-stand) and with Namibian German patterns (NAM-lex and NAM-gram)? RQ2) How does the linguistic domain (NAM-lex vs. NAM-gram) influence the potential to evoke social meaning? RQ3) How are identity constructions related to language attitudes, i.e., identity-related evaluations such as solidarity and place of origin and residence?

With respect to RQ1, our findings show that DE-stand was associated with high competence and with Germany as the speaker’s place of residence and origin, whereas NAM-lex features were associated with higher solidarity and Namibia as the place of residence and origin. The factor analysis confirmed that four of the five items on each dimension clustered together. The differential *intelligent* was not part of the competence cluster, possibly because both speakers were rated highly with regard to intelligence across all conditions. The reason that the differential *humorous* was not part of the solidarity cluster might be that speakers were evaluated as less humorous in the NAM-gram condition than in the DE-stand condition. Nevertheless, the general pattern confirms the expected association between, on the one hand, standard language variety and high competence and, on the other hand, ingroup non-standard variety and high solidarity (Trudgill, 1986; Preston and Robinson, 2005; Bekker, 2019; Dragojevic et al., 2021). Moreover, DE-stand and NAM-lex were associated with different situations where Namibian German speakers usually use those varieties, i.e., Standard Germany German in professional contexts and with tourists, and Namibian German in interactions with friends and in relaxed situations (see Leugner, 2023; Wiese et al., 2022 for those usage contexts). DE-stand was less associated with Germany as a place of origin (in comparison to place of residence), probably because Namibian German speakers learn Standard German in school settings and use it in interactions with tourists or during visits to Germany (Shah and Zappen-Thomson, 2018). Interestingly, place of living and origin interacted with competence and solidarity measures, suggesting that the expected social group had a strong impact on ratings: Speakers regarded as in/from Germany, i.e., belonging to the outgroup who speak the prestigious standard, were considered as “competent” speakers even if they used NAM-lex features. In addition, their solidarity ratings were not influenced by language variety, possibly because Namibian German is spoken neither in Germany nor by Germans. However, evaluations of speakers regarded as in/from Namibia, i.e., ingroup members, were influenced by language variety: they only received high solidarity ratings if they used NAM-lex features, and high competence ratings if they spoke DE-stand. Notably, as pointed out by a reviewer, Namibian-lexical features may differ in social meaning depending on whether they are borrowings from English or Afrikaans due to the historical context of Namibia. Indeed, previous research showed Namibian German (younger) speakers associate English with “globalization” and “urbanity” while Afrikaans is associated with “backwardness” and the “countryside” (Leugner, 2023). Further research differentiating English versus Afrikaans lexical borrowings may clarify how these associations influence competence and solidarity ratings.

With respect to RQ2, the results confirm that the linguistic domain influences the activation of stereotypes and social meaning. Namibian German grammatical features were not strongly associated with high competence or solidarity, but rather occupied an interesting intermediate position: they pattern with Nam-lex with respect to competence, but with DE-stand with respect to solidarity. In addition, NAM-gram features were not strongly associated with Namibia as a place of residence, even though they were slightly more associated with Namibia as a place of origin. This weaker association, compared to NAM-lex complements previous research on the impact of the linguistic domain on language stereotype activation (Labov, 2001; Matras, 2009; Meyerhoff and Walker, 2013; Levon and Buchstaller, 2015) and supports the suggestion that grammatical features may have less social salience, i.e., a weaker potential to evoke social meaning (Wiese et al., 2022). Additionally, they may be more ambiguous because they would usually co-occur with Namibian-specific lexical features in real-life situations, and because some of the grammatical features may also occur in non-standard varieties in Germany, i.e., varieties that Namibian German speakers may be exposed to by social media from Germany, visits to Germany, and in school due to teachers from Germany. Both interpretations are compatible with the comments volunteered by participants, who variably associated the NAM-gram stimuli with attempts both to speak Standard German and to speak Namibian German. The interpretation of attempts (rather than successes) may reflect that in cases of ambiguity, listeners may consciously or unconsciously not associate a speaker either with high solidarity or with high competence, regardless of the speaker’s assumed origin or place of residence.

These findings also relate to RQ3, the relation between language use and identity construction. Only the use of NAM-lex features was a strong ingroup index, as reflected by high solidarity ratings and the association with Namibia and with relaxed situations with friends (i.e., ingroup members). However, NAM-lex features did not influence solidarity ratings if Germany was assumed as the place of origin and residence, that is, if the speaker was regarded as an outgroup member or as living in a context where Namibian German (ingroup) solidarity is usually not expressed. DE-stand was regarded as an index of Germany and Germans and associated with higher competence ratings. This means that positive competence evaluations were not restricted to the ingroup (Namibian Germans), but also occurred for the outgroup (Germans), probably because of the social prestige granted to Standard Germany German.

Taken together, the findings agree with suggestions that the German-speaking minority uses different varieties to negotiate identity in the special multilingual setting in Namibia (Wiese et al., 2022; Leugner, 2023): German in general can index belonging to the German-speaking minority in Namibia and demarcation from other speaker groups in its multilingual society, whereas Namibian German can specifically index belonging to Namibia and demarcation from German tourists (Wiese et al., 2022). Notably, it is the societal setting of Namibia, with its appreciation of multilingualism and (ethno-linguistic) diversity as well as the status of German as the language of a privileged minority group, where German has been endowed with the status of one of the national languages, that allows this negotiation of different identities. In this setting, German can be maintained as a vital heritage language (Wiese et al., 2017), in stark contrast to monolingually oriented societal contexts like, e.g., the US or Australia, where German as a minority language with low prestige and market value is usually in decline and is not maintained systematically over generations (Plewnia and Riehl, 2018, see also Dragojevic et al., 2013).

Interestingly, the high prestige of the Standard Germany German variety reflects that standard language ideology with respect to the heritage language is also present in a minority group in such a multilingually oriented setting. In general, language stereotypes and ideologies are acquired from an early age (Kinzler et al., 2007; Kinzler and DeJesus, 2013), in particular through exposure to media and in school settings (Dragojevic et al., 2013; Gogolin, 2008; Lippi-Green, 1997). In our setting, they are acquired within the German-speaking minority and probably fostered by the school setting, including the contact with German school teachers at private schools, and by contact with Germany (and the language ideologies held there) via German media input, tourists, and visits to Germany.

Both factors are difficult to disentangle, given that language ideologies also play a role in multilingual contexts beyond the German-speaking minority in Namibia. Fostered by hegemonial structures and colonial history, languages (varieties) in multilingual societies differ in their prestige or market value, which influences the social meaning associated with them (Giles and Watson, 2013), their usage in inter- and intraethnic communication and in identity construction (Stell and Dragojevic, 2017), and these language attitudes are acquired early (Kinzler et al., 2012). For further research, it would be interesting to systematically and comparatively investigate these factors, for instance, by taking into account languages (e.g., Pacific languages) and societies in which such hierarchical ideologies and, in particular, standard language ideology may not play such a strong role (Dragojevic et al., 2013; Schneider, 2025).

## 6 Conclusion

We report an open-guise study that examined how linguistic choice in different linguistic domains evokes social meaning in Namibian German speakers. Standard Germany German was associated with high competence ratings and Germany as the speaker’s place of origin and residence, but also with communicative situations in Namibia where competence in Standard German is relevant. Lexical Namibian German features (borrowings from English) evoked high solidarity ratings, lower competence ratings, and Namibia as the speaker’s place of origin and residence, and were associated with communicative situations in Namibia where ingroup membership is salient. Grammatical Namibian German features (non-standard grammatical patterns of German found in Namibia) were associated with Namibia as a place of origin, but with both Namibia and Germany as places of residence, and with lower solidarity and competence ratings. The competence associated with Standard Germany German reflects the high status and economic values of (standard) German among the Namibian German minority in Namibia and the impact of standard language ideology, while the solidarity associated with Namibian German reflects the use of Namibian German to index ingroup membership with the Namibian German minority in Namibia and demarcation from German tourists. These results support earlier findings on an association of standard language use with competence and prestige and non-standard ingroup varieties with solidarity, adding evidence from a multilingually oriented society. Furthermore, they highlighted the intermediate status of grammatical non-standard characteristics compared to, on the one hand, standard language and, on the other hand, lexical characteristics. Above and beyond that, the Namibian context allowed us to target patterns of identity construction and language use in a societal macro context where (ethno-) linguistic diversity is acknowledged and appreciated, and where, accordingly, German, as a minority language, nevertheless has the status of one of the national languages and enjoys a high vitality in its speech community. Our study showed that in such a context, the negotiation of ethnolinguistic identities between German ethnicity and Namibian belonging can tap into a rich source of linguistic options, including standard language as well as ingroup registers.

## Data Availability

The original contributions presented in the study are included in the article/Supplementary material, further inquiries can be directed to the corresponding author.
