# Supplementary material for: Negotiating ethnolinguistic identity in a multilingual society: social meaning and linguistic choice in Namibian German
Source: Front Psychol. 2026 May 29;17:1819162. doi: 10.3389/fpsyg.2026.1819162 (PMC13259862; doi:10.3389/fpsyg.2026.1819162)
Supplement: Supplementary file 1 [file Data_Sheet_1.pdf]

Supplementary materials

DE-stand

Hallo! Ähm, tut mir leid, dass ich zu *spät* bin. Also ich war vorhin *einkaufn* un als ich fertich war, is da n *Unfall* passiert. Ich hab da aufm *Parkplatz* gestandn un da war da sone ältre *Frau*, die is da mit ihrm Einkaufswagn lang gelaufn. Ich glaub nich, dass die wirklich *aufgepasst hat*. Die war an ihrm *Handy*. Und auf eima ist da n *Auto* von hintn gekomm. Der Typ im Auto hat die Frau glaubich nich *gesehn* un hat se einfach *angefahrn*. Da ist die *hingefalln* un hat sich erschrocken, weil sie keine Luft bekam. Aber der Fahrer is nich *ausgestiegn*, ne? Der is einfach im *Auto* sitzn gebliebm. Dann is aber n *andrer* Typ gekomm un hat ihr *aufgeholfn*. Einklich is nich viel *passiert*, die Frau hat sich zum Glück nich doll *wehgetan*. Ich bin auch nomma zu ihr *hingegang*, aber mit ihr war alles in *Ordnung,* un sie hat keine Hilfe gebraucht. Aber das hat mich *aufgehaltn* un deswegn war ich spät *dran*.

NAM-gram

Hallo! Ähm, tut mir leid, dass ich *spät* bin. Also ich war vorhin *einkaufn* un als ich fertich war, is da n *Unfall* passiert. Ich hab da aufm *Parkplatz* gestandn un da war da sone ältre *Frau*, die is da mit ihrm Einkaufswagn lang gelaufn. Ich glaub nich ,die *hat* wirklich aufgepasst. Die war an ihrm Handy. Und auf eima n *Auto* is da von hinten gekomm. Der Typ im Auto hat die Frau glaub ich nich gesehn un hat se einfach angefahrn. Da ist die hingefalln un hat sich erschrocken, weil sie keine Luft bekam. Aber Fahrer is nich ausgestiegn. Der is einfach im Auto sitzn gebliebm. Dann is aber n andrer Typ gekomm un hat ihr aufgeholfn. Einklich is nich viel passiert, die Frau hat zum Glück nich doll *weh* gekricht. Ich bin auch nomma zu ihr hingegang, aber mit ihr war alles in Ordnung, un sie hat keine Hilfe gebraucht. Aber das hat mich aufgehaltn un deswegn war ich spät dran.

NAM-lex

Hallo! Ähm, tut mir leid, dass ich *spät* bin. Also ich war vorhin *einkaufn* un als ich fertich war, is da n *Unfall* passiert. Ich hab da aufm *Parkplatz* gestandn un da war da sone ältre *Frau*, die is ist da mit ihrm Trolly lang gelaufen. Ich glaub nich, die hat wirklich aufgepasst. die war an ihrm *Phone* . Und auf eima is da n Auto von hinten gekomm. Der Typ im Auto hat die Frau glaubich nich *gesehn* un hat se einfach angefahrn. Da ist die hingefalln, un hat gepanikt, weil sie keine Luft bekam. Aber der Fahrer ist nicht ausgestiegen. Der is einfach im *Auto* sitzn gebliebm. Dann is aber n andrer Typ gekomm un hat ihr aufgeholfn. Eigentlich is nich viel passiert, die Frau hat sich zum Glück nich doll wehgetan. Ich bin auch nomma zu ihr hingegang, aber mit ihr war alles *oreit,* un sie hat keine Hilfe gebraucht. Aber das hat mich aufgehaltn un deswegn war ich spät dran.

English translation

Hello there! Um, I'm sorry I'm late. So I was shopping earlier and when I was done, there was an accident. I stood there in the parking lot and there was an older woman who walked there with her shopping cart. I don't think she really paid attention. She was on her phone. Once upon a time, a car came from behind. The guy in the car I don't think I saw the woman and just hit her. She fell down and was frightened because she couldn't breathe. But the driver didn't get out, did he? He just stayed in the car. But then another guy came and helped her out. Actually, not much happened, the woman fortunately did not hurt so much. I went to see her again, but everything was fine with her, and she didn't need any help. But that stopped me and that's why I was late.
