# Supplementary material for: Negotiating ethnolinguistic identity in a multilingual society: social meaning and linguistic choice in Namibian German
Source: Front Psychol. 2026 May 29;17:1819162. doi: 10.3389/fpsyg.2026.1819162 (PMC13259862; doi:10.3389/fpsyg.2026.1819162)
Supplement: Supplementary file 3 [file Data_Sheet_3.PDF]

```
30 variables, 3 rows per participants
variables:
Part_ID            : Participant_ID (1-35)
Stim_Gender        : gender of the Speaker ("female" vs. "male")
Condition          : experimental condition ("DE-stand" vs. "NAM-gram" vs. "NAM-lex")
Presentation_Order: order of the presentation of the experimental Stimuli ("sd-lex-gram", "gram-lex-sd", etc.)
Likeable           : semantic differential rating on a scale 1-7
Ambitious          : semantic differential rating on a scale 1-7
Humorous           : semantic differential rating on a scale 1-7
Self_confident     : semantic differential rating on a scale 1-7
Relaxed            : semantic differential rating on a scale 1-7
Successful         : semantic differential rating on a scale 1-7
Competent          : semantic differential rating on a scale 1-7
Friendly           : semantic differential rating on a scale 1-7
Familiar           : semantic differential rating on a scale 1-7
Intelligent        : semantic differential rating on a scale 1-7
EstimatedAge       : estimated age of the speaker (as provided of the participant)
ResidenceOriginal : expected place of residence of the speaker (as provided of the participant)
ResidencePrecoded : precoding of the expected place of residence
OriginPrecoded     : precoding of the expected place of residence
OriginOriginal     : expected place of origin of the Speaker (as provided of the participants)
SituationPrecoded : precoding of the epected situation or place where to meet the Speaker
SituationOriginal : epected situation or place where to meet the Speaker (as provided of the participants)
AdditionalCharacteristics: additional characteristics of the speaker (provided by the participants)
Part_Age           : age of participant
Part_Gender        : gender of participant
Part_Raised        : place where participant grew up
F_Solidarity       : combinded solidarity measure (based on statistical analysis)
F_Competence       : combinded competence measure (based on statistical analysis)
Residence          : place of residence variable as analysed in the paper (Germany vs. Namibia vs. other)
Origin             : place of origin variable as analysed in the paper (Germany vs. Namibia vs. other)
Situation          : situation variable as analysed in the paper (education vs. farm vs Germany vs.
job/authorities vs. leisure s. public (Namibia) vs. tourism vs. visits vs. unspecific)
```
